# The Impact of Maltitol-Sweetened Chewing Gum on the Dental Plaque Biofilm Microbiota Composition

**DOI:** 10.3389/fmicb.2018.00381

**Published:** 2018-03-06

**Authors:** Bart J. F. Keijser, Tim J. van den Broek, Dagmar E. Slot, Lodewic van Twillert, Jolanda Kool, Clémentine Thabuis, Michel Ossendrijver, Fridus A. van der Weijden, Roy C. Montijn

**Affiliations:** ^1^Microbiology and Systems Biology Group, Netherlands Organisation for Applied Scientific Research, Zeist, Netherlands; ^2^Department of Preventive Dentistry, Academic Center for Dentistry Amsterdam, University of Amsterdam, Amsterdam, Netherlands; ^3^Department of Periodontology, Academic Center for Dentistry Amsterdam, University of Amsterdam and VU University Amsterdam, Amsterdam, Netherlands; ^4^Biology and Nutrition Department, Roquette Frères, Lestrem, France

**Keywords:** oral health, oral biofilm, supragingival plaque, microbiota, maltitol, polyol, *Actinomyces*

## Abstract

**Background:** The oral cavity harbors a complex microbial ecosystem, intimately related to oral health and disease. The use of polyol-sweetened gum is believed to benefit oral health through stimulation of salivary flow and impacting oral pathogenic bacteria. Maltitol is often used as sweetener in food products. This study aimed to establish the *in vivo* effects of frequent consumption of maltitol-sweetened chewing gum on the dental plaque microbiota in healthy volunteers and to establish the cellular and molecular effects by *in vitro* cultivation and transcriptional analysis.

**Results:** An intervention study was performed in 153 volunteers, randomly assigned to three groups (www.trialregister.nl; NTR4165). One group was requested to use maltitol gum five times daily, one group used gum-base, and the third group did not use chewing gum. At day 0 and day 28, 24 h-accumulated supragingival plaque was collected at the lingual sites of the lower jaw and the buccal sites of the upper jaw and analyzed by 16S ribosomal rRNA gene sequencing. At day 42, 2 weeks after completion of the study, lower-jaw samples were collected and analyzed. The upper buccal plaque microbiota composition had lower bacterial levels and higher relative abundances of (facultative) aerobic species compared to the lower lingual sites. There was no difference in bacterial community structure between any of the three study groups (PERMANOVA). Significant lower abundance of several bacterial phylotypes was found in maltitol gum group compared to the gum-base group, including *Actinomyces massiliensis* HOT 852 and *Lautropia mirabilis* HOT 022. Cultivation studies confirmed growth inhibition of *A. massiliensis* and *A. johnsonii* by maltitol at levels of 1% and higher. Transcriptome analysis of *A. massiliensis* revealed that exposure to maltitol resulted in changes in the expression of genes linked to osmoregulation, biofilm formation, and central carbon metabolism.

**Conclusion:** The results showed that chewing itself only marginally impacted the plaque microbiota composition. Use of maltitol-sweetened gum lowered abundance of several bacterial species. Importantly, the species impacted play a key role in the early formation of dental biofilms. Further studies are required to establish if frequent use of maltitol gum impacts early dental-plaque biofilm development.

## Introduction

The oral cavity is a complex ecosystem, harboring one of the most diverse microbial populations found in the human body (Human Microbiome Project Consortium, 2012). Within the mouth, distinct niches can be discerned, characterized by unique biochemical and physical properties, which is reflected in the microbial community structures that occupy these various niches (Duckworth, 2006). Over 700 species have been identified spanning more than 20 phyla (Zaura et al., 2009; Dewhirst et al., 2010). Microorganisms residing in the mouth are required to form biofilms and firmly attach to the various surfaces provided. This includes soft mucosal tissues of the gums, cheeks, and tongue and the hard tissues of the teeth. Oral biofilms have been shown to form intricate multispecies structures occasionally described as *corn cobs*, *hedgehogs*, or *cauliflowers*, providing optimal biochemical conditions for the different members within, protecting against abiotic conditions and allowing an optimal concerted metabolism (Mark Welch et al., 2016).

Surface attachment, nutrient availability, and biophysical properties (e.g., oxygen and pH levels) appear to be the main driving forces in the selection of microbial populations (Proctor and Relman, 2017; Zaura et al., 2017). Dysbiotic shifts in the composition of the commensal microbiota have been proposed as the underlying cause in the onset and progression of oral diseases, including dental caries and gingival inflammatory diseases (Sanz et al., 2017).

The use of sugar-free chewing gum as an adjunct to tooth brushing provides a small but significant reduction in plaque scores (Keukenmeester et al., 2013). Chewing gum has also been shown to have beneficial effects in the prevention of dental caries by increasing the flow of saliva (Dodds, 2012). Sugar-free chewing gum is often sweetened by polyol sugars such as xylitol, sorbitol, maltitol, and erythritol (Livesey, 2003). Unlike regular sugars, polyol alcohols cannot be used by oral bacteria for fermentation, are not metabolized to organic acids, and thus do not contribute to tooth decay (Matsuo, 1973; Van Loveren, 2004).

It has been demonstrated both *in vivo* as well as *in vitro* that xylitol and sorbitol inhibit the growth of a number of cariogenic bacteria, including *Streptococcus mutans* and *Streptococcus sobrinus* (Soderling et al., 2011; Thabuis et al., 2013; Haghgoo et al., 2015; Makinen, 2016). The mechanism of action is assumed to be due to an accumulation of the sugar alcohol within the cell upon uptake, resulting in the formation of a toxic sugar-phosphate (Trahan et al., 1996; Miyasawa-Hori et al., 2006). The effects of polyol-sweetened gums on the healthy oral microbiota have not been established.

This study aims to establish the effects of frequent consumption of maltitol-sweetened chewing gum on the supragingival plaque microbiota composition and to gain insights in the effects of maltitol at a molecular and cellular level.

## Materials and Methods

### Ethical Aspects

This study was carried out in accordance with the recommendations of the ethical review board of the Academic Centre for Dentistry Amsterdam. The protocol was independently reviewed and approved by the Medical Ethics Committee of the Academic Medical Centre Amsterdam under number MEC NL45518.018.13 and registered at the Dutch Trial Register (NTR4165). All subjects gave written informed consent in accordance with the Declaration of Helsinki. The clinical study was conducted approximating Good Clinical Practice (GCP) as laid out in the International Conference on Harmonization (ICH). The study was scheduled and executed from September–December 2013 at the Department of Periodontology at the Academic Centre for Dentistry Amsterdam, Netherlands. Participants were recruited from a database that contained individuals from universities in and around Amsterdam who had subscribed as being potentially interested in participating in clinical research. Flyers, posters, and advertisements were used to attract additional participants. Those participants who responded were informed about the research by a recruitment letter sent by e-mail. All voluntary participants were informed about the outline, purpose, and duration of the study. Subsequently, participants had time to consider whether they wished to be involved and undergo screening. Participants were asked to read and sign the informed consent form, and they received a signed copy for their records.

### Randomization and Allocation Concealment

This study had a parallel single-blind (examiner) design with three randomly assigned groups. It was designed to evaluate the effect of the frequent use of maltitol chewing gum during 28 days on the supragingival microbiota. Block randomization was performed by the study coordinator using true random numbers, which were generated by sampling and processing a source of entropy outside the computer^1^. The principal investigator was responsible for allocation concealment. No stratification was applied.

The examiners were blinded to the treatment randomizations, and records of earlier examinations were not available at each re-examination. Instruction took place in an area separate from the examiners by the coordinator. The randomization code was kept in a sealed envelope that was not accessible to the examiners. Participants were firmly instructed not to reveal their product assignment to the examiners.

### Study Population

For the present study, 153 systemically healthy participants were recruited from non-dental students. The inclusion criteria were being ≥18–45 years of age, non-smokers (Lie et al., 1998), possessing at least 20 natural teeth (minimum of five evaluable teeth per quadrant), and being considered systemically healthy as assessed by a medical questionnaire. At clinical screening, participants were examined for a full-mouth bleeding on marginal probing (BOMP) score (Lie et al., 1998) of 30–60% to be included.

The exclusion criteria were overt dental caries and periodontitis. The latter was clinically assessed according to Dutch Periodontal Screening Index (DPSI) with excluding scores 3+ and 4 (Mantilla Gomez et al., 2001; Van der Velden, 2009). Additional exclusion criteria were orthodontic appliances, removable (partial) dentures, night guards, oral and/or peri-oral piercings, apparent oral lesions, use of antibiotics in the preceding 2 months, pregnancy and breast feeding. Eligible participants did not use any interdental device as part of their daily oral hygiene procedure and had not participated in a clinical trial in the previous 30 days.

After meeting all study-entrance criteria, participants were considered eligible for the study and scheduled for the two appointments (each visit at the same time on the same day of the week).

### Study Products and Procedures

The products were provided and their use was individually instructed by one and the same study coordinator. Standardized instructions were given to the participants. In the 15 days preceding the first appointment (day 1), participants were asked to refrain from using any kind of polyol-containing food products, including chewing gum and lozenges. From the screening visit onward, participants were instructed to brush twice daily (morning and evening) in their customary manner with a standard toothbrush and standard toothpaste as provided (Tubes of 75 ml with 1450 ppm sodium fluoride; HEMA^®^ toothpaste, HEMA B.V., Netherlands). Furthermore, they were instructed not to use any interdental cleaning device or a mouthwash during the trial period. At day 1 subjects were instructed to comply with the following instructions adapted according to their group assignment (maltitol test gum, gum base, or no gum).

Instructions for maltitol gum use were to chew 10 pieces of gum daily, 2 pieces at the same time, 5 times daily for 10 min (Maltitol chewing gum provided by Roquette Frères). Instructions for gum base use were to chew 5 pieces of gum daily, 5 times daily, 1 piece for 10 min (gum base provided by Roquette Frères). In both the maltitol gum and gum-base groups, volunteers were instructed to use gum after breakfast, lunch, dinner in the afternoon, and before going to bed.

All three groups were requested to refrain from brushing for 24 h before each appointment while the gum groups were asked to continue their chewing assignment. Participants were obliged to note in a customized calendar the moment of brushing and, if applicable, the moment of chewing gum intake.

### Sample Collection and Processing

At day 0, 28, and 42, upper-buccal and lower-lingual supragingival plaque was collected scraping the surfaces of molars, premolars, cuspid, and incisors with a sterile Teflon spatula from two randomly chosen contra-lateral quadrants (one in the upper jaw, one in the lower jaw). Sampling was performed by the same examiner during the study (EJCM). Plaque was transferred to an Eppendorf vial with 50 μl RNAprotect solution, spun down using a microcentrifuge and stored on ice until transport to the laboratory and then stored at -80°C until further processing for DNA extraction.

### Microbiota Analysis

For DNA isolation, plaque samples were thawed on ice and lysed by bead beating (Mini-BeadBeater-24; BioSpec Products, Bartlesville, OK, United States) for 2 min at 2100 oscillations/min in the presence of 300 μl of lysis buffer (Mag Mini DNA Isolation Kit; LGC Ltd., United Kingdom), 500 μL zirconium beads (0.1 mm; BioSpec Products, Bartlesville, OK, United States) and 500 μL phenol saturated with 10 mM Tris–HCl and 1 mM EDTA pH 8.0 (Sigma). After centrifugation, DNA was extracted using the Mag Mini DNA Isolation Kit (LGC Ltd., United Kingdom) in accordance with the manufacturer’s instructions.

To determine the amount of bacterial DNA, a quantitative polymerase chain reaction (qPCR) was performed using primers *16Suni-I-F* CGA AAG CGT GGG GAG CAA A, *16Suni-I-R* GTT CGT ACT CCC CAG GCG G, and 6-FAM MGB probe ATT AGA TAC CCT GGT AGT CCA that are specific for the bacterial 16S rRNA gene. In the reaction, 1 μl of a 10-fold diluted DNA sample is added to 24 μl of a mixture of primers and RT PCR master mix (Diagenode, Seraing, B) and is analyzed on an Applied Biosystems 7500 RT PCR system during 40 cycles of denaturation at 95.0°C for 15 s and annealing/elongation at 60.0°C for 1 min. A standard curve was established by analysis of a pooled DNA sample serially diluted at 1 ng, 100 pg, 10 pg, 1 pg, 100 fg, 10 fg, and 1 fg per μl as well as a negative control. After conversion of the *C*t values to DNA quantities, the number of cells was estimated by accounting for the elution volume used after DNA extraction (60 μl) and assuming that 1000 fg of DNA was equivalent to 447.4 cells, i.e., a genome size of 2 Mb.

For 16S rDNA amplicon sequencing of the V4 hypervariable region, 1 ng of DNA was amplified as described by Kozich et al. (2013) with the exception that 30 cycles were used instead of 35, using F515/R806 primers (Caporaso et al., 2011). Primers included the Illumina adapters and a unique 8-nt sample index sequence key (Kozich et al., 2013). The amount of DNA per sample was quantified using the dsDNA 910 Reagent Kit on the Fragment Analyzer (Advanced Analytical). The amplicon libraries were pooled in equimolar amounts and purified using the Gel Extraction Kit (Qiagen). The Library was quantified using the Quant-iT^TM^ PicoGreen^®^ dsDNA Assay Kit (Thermo Fisher Scientific). Paired-end sequencing of amplicons was conducted on the Illumina MiSeq platform (Illumina, Eindhoven, Netherlands). The sequence data was processed with mothur v.1.31.2 (Schloss et al., 2009) in line with the mothur MiSeq SOP (Kozich et al., 2013). After even sampling at 3,000 sequences, the sequences were grouped in operational taxonomic units by Minimal Entropy Decomposition (MED) (Eren et al., 2015) using a minimum substantive abundance value (-M) of 9. Taxonomy was then assigned by querying the representative sequence of each oligotype against the Human Oral Microbiome Database (HOMD) RefSeq v.13.2^2^ (Chen et al., 2010) using the naïve Bayesian classifier. Sequence data is available through the NCBI sequence read archive (SRA) under accession number SUB3627297.

### Statistical Methods

Statistical analysis and data visualization was performed in R (R Development Core Team, 2016, version 3.3.2) using packages ggplot2 (version 2.2.1) (Wickham, 2009), vegan (version 2.4-1) (Oksanen et al., 2013), and glmmADMB (version 0.8.3.3) (Fournier, 2012).

Multi-dimensional Scaling (MDS) was performed using parts of the vegan package, while negative binomial regression (nbreg) was performed using the glmmADMB package.

MDS were performed using the Bray–Curtis distance measure. When applicable, nbreg was performed with subject as a random factor. For nbreg, the overdispersion parameter α was estimated automatically.

### Microbiological Cultivation Studies

*In vitro* cultivation studies were performed using the following *Actinomyces* isolates: *A. naeslundii* (DSM-43013), *A. oris* (DSM-23056), *A. johnsonii* (DSM-23038), *A. massiliensis* (DSM-23047), *A. gerencseriae* (DSM-6844), *A. dentalis* (DSM-19115), and *A. israelii* (DSM-43320). Isolates were grown anaerobically in Schaedler Anaerobe Broth (SAB) medium in the presence of 0, 0.25, 0.5, 1, and 2% Maltitol. Cultivations were done in triplicate. Qualitative growth was assessed daily by visual inspection. Quantitative growth was assessed after 7 days of incubation by qPCR using primers 16S-Actmycs-gr-F 5′-GGGTTGTGAACCTCTTTCGCC-3′, 16S-Actmycs-gr-R 5′-GCTGGCACGTAGTTAGCCG-3′, and Taqman MGB probe 16S-Actmycs-gr 5′-TGTGGKKGGGTTGACG-3′. qPCR was performed in RT PCR master mix (Diagenode, Seraing, B) on an Applied Biosystems 7500 RT PCR system during 45 cycles of with a denaturation step at 95.0°C for 15 s and an annealing/elongation step at 60.0°C for 1 min. A standard curve was established by analysis of an *A. massiliensis* genomic DNA sample, serially diluted at 2 ng, 200 pg, 20 pg, 2 pg, 200 fg, 20 fg, and 2 fg per μl as well as a negative control.

### Transcription Analysis

*Actinomyces massiliensis* (DSM-23047) was precultured anaerobically in 10 ml SAB medium at 37°C for 4 days. Next, the *A. massiliensis* preculture was diluted 1000× in SAB medium and cultivated for 4 days. For the exposure, the spent medium was carefully taken off and replaced by fresh prewarmed SAB and SAB with 2% Maltitol (Sigma M8892). After 1 h of incubation, cells were harvested and quenched in liquid nitrogen. For RNA isolation, bacterial cells were lysed by phenol bead beating and purified using the ChargeSwitch RNA total kit (Invitrogen 45-7006) in accordance with the manufacturers recommendations. RNA integrity was assessed on the fragment analyzer. mRNA was enriched by the Ribo-Zero^®^ rRNA Removal Kit (Illumina). Following mRNA enrichment, the samples were cleaned up using the Agencourt RNAClean XP kit (cat no. A63987, Beckman Coulter): Libraries were prepared by the TruSeq Stranded mRNA HT library kit (Illumina, RS-122-2103) according to the manufacturer’s recommendations. Sequencing was performed in the Illumina NextSeq 500.

Clean data were obtained from the raw data by removing the sequences of the adapters and low-quality reads using Btrim64-static (Kong, 2011). The clean reads were aligned to the *A. massiliensis* F0489 genome (GenBank accession number: AKFT00000000) using STAR with default parameters, allowing up to one-base mismatches (Dobin et al., 2013). Sequence count tables were created with HTSeq (Anders et al., 2015). Genes of which expression values were 0 in all samples were removed from the dataset before exploratory analysis. Data were normalized and differentially expressed genes (DEGs) between control and maltitol-treated samples were extracted by using DESeq2 (Love et al., 2014) with default options, including log fold change shrinkage. The DEGs were defined with a false discovery rate (FDR) ≤ 0.05.

## Results

### Intervention Study

The intervention study had a parallel single-(examiner)blind design with three randomly assigned groups using block randomization, each consisting of approximately 50 participants. **Supplementary Figure S1** is a flow diagram that represents the passage of the patients through this clinical trial. Following recruitment and a standardized run-in phase, volunteers were divided into three groups. One group that was requested to use five times daily maltitol gum. The second group used gum base. The third group did not use any chewing gum. Throughout the run-in phase and the intervention study, volunteers were provided a standardized toothbrush and toothpaste and were requested to refrain from using any kind of polyol containing food products, including chewing gum and lozenges. During the study, eight participants reported adverse events. Four cases were unrelated to the study products, three possibly related (gastro-intestinal complaints or pain due to chewing on the occlusal lesion), and one was related as an undiagnosed flatulence and itch ever since using the study products.

### Total Bacterial Load in Dental Plaque

Supragingival plaque was collected from two randomly chosen contra-lateral quadrants (one in the upper jaw and one in the lower jaw). At baseline (day 0) and at day 28, the lower jaw lingual and the upper jaw buccal surfaces of molars and premolars were sampled using a sterile Teflon spatula. The lower jaw supragingival plaque was also sampled at day 42, 2 weeks after completion of the intervention. After extraction of DNA, the total bacterial load was determined by 16S rDNA qPCR. Results are shown in **Table 1**. The bacterial density of the lingual plaque samples of the lower jaw was found to be higher than that of the buccal plaque samples of the upper jaw. The bacterial load of the lower jaw lingual plaque samples at baseline was on average 6.80 × 10^8^ (σ 6.53 × 10^8^) cells and for the upper jaw buccal plaque was on average 1.22 × 10^8^ (σ 9.21 × 10^7^) cells. There was no significant change in the total bacterial load of the lower jaw lingual plaque in any of the three study groups when comparing baseline with the day 28 samples. For the upper-jaw buccal plaque samples, however, a significant decrease in bacterial load was observed of ∼80% for all three groups, showing no significant difference between groups. A moderate-to-strong correlation (Pearson correlation ρ = 0.66) was found in the total bacterial load of lower-jaw lingual plaque samples of the participants when comparing the baseline with the day-28 samples. The bacterial load of upper-jaw buccal plaque samples showed a weak correlation (*r* = 0.44) between these two time points.

**Table 1:** Total bacterial load (Cells/sample), as established by broad range 16S rDNA qPCR of supragingival plaque samples.

Total bacterial load	Upper-jaw buccal plaque		Lower-jaw lingual plaque		
		
	D0	D28	D0	D28	D42
Control	1.13 × 10^8^	2.18 × 10^7^	7.40 × 10^8^	5.75 × 10^8^	3.78⋅10^8^
	(9.92 × 10^7^)	(3.13 × 10^7^)	(6.19 × 10^8^)	(6.02 × 10^8^)	(4.06 × 10^8^)
Gum base	1.13 × 10^8^	2.03 × 10^7^	6.16 × 10^8^	5.32 × 10^8^	3.02 × 10^8^
	(8.14 × 10^7^)	(2.75 × 10^7^)	(4.85 × 10^8^)	(6.42 × 10^8^)	(2.88 × 10^8^)
Maltitol	1.38 × 10^8^	2.93 × 10^7^	6.84 × 10^8^	7.70 × 10^8^	3.60 × 10^8^
	(2.93 × 10^7^)	(4.10 × 10^7^)	(5.50 × 10^8^)	(7.83 × 10^8^)	(3.43 × 10^8^)


**Shown is the average value and between brackets the standard deviation for each study group at each time point [day 0, 28, and 42 (lower jaw lingual plaque)].**

### The Microbiota of Buccal and Lingual Supragingival Plaque Differs

**FIGURE 1:**
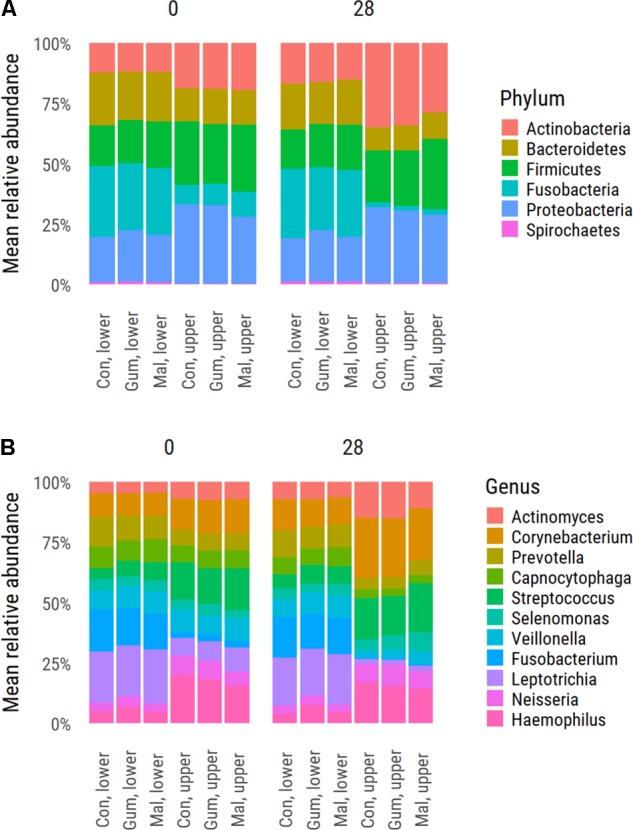
Average distribution values of the supragingival plaque microbiota at phylum **(A)** and genus level **(B)**. Shown is the distribution for each study group at baseline d0 and upon completion of the trial (day 28).

The supragingival plaque microbiota composition of the upper-jaw buccal and lower-jaw lingual samples was analyzed by sequencing 16S rDNA amplicons for all 153 participants at baseline (day 0) and at day 28 as well as at day 42 for the lower-jaw lingual samples. In total, 9,717,143 sequences remained after quality filtering and even sampling. The lower-jaw lingual plaque microbiota had a small but statistically significant higher diversity index as compared to that of the upper-jaw buccal plaque samples. The Shannon diversity indices for upper buccal and lower lingual plaque samples were 5.09 (σ = 0.487) and 4.96 (σ = 0.581), respectively. Phylum level comparison of upper buccal and the lower lingual supragingival plaque at baseline (day 0) revealed significant differences in composition between these two sites (**Figure 1A**). The upper buccal plaque microbiota was dominated by members of the phylum Proteobacteria (30.9%), Firmicutes (26.4%), Actinobacteria, (19.2%) Bacteroidetes (14.0%), and Fusobacteria (9.0%). Lower-lingual plaque was dominated by Fusobacteria (28.2%), Bacteroides (20.6%), Proteobacteria (19.8%), Firmicutes (18%), and Actinobacteria (12.0%). Dominant genera in the upper-buccal plaque samples were *Haemophilus*, *Streptococcus*, *Corynebacterium*, and *Neisseria*. The lower-lingual plaque samples were dominated by *Leptotrichia*, *Fusobacterium*, *Prevotella*, *Veillonella*, and *Capnocytophaga* (**Figure 1B**). To further delineate the taxonomic differences between upper-buccal and lower-lingual plaque microbiota, we used MED and taxonomically assigned representative sequences using the naïve Bayesian classifier and the HOMD as reference. Significant differences between lower-jaw lingual and upper-jaw buccal plaque at baseline were identified through nbreg analysis of the baseline data with jaw as a fixed factor and subject as random factor. Significant differences were linked to an over-abundance of MED nodes assigned as *Streptococcus gordonii*, HOT 055, and HOT 056 *Granulicatella adiacens* (paraadiacens), HOT 534, *Gemella morbillorum*, HOT 046, residing in the upper buccal supragingival plaque. The lower-lingual plaque was found to be dominated by *Fusobacterium nucleatum* ss_vincentii, HOT 200, *Tannerella* sp., HOT 286, *Catonella* sp., HOT 164, *Dialister invisus*, HOT 118, and a number of *Leptotrichia* species, including *Leptotrichia hongkongensis*, HOT 213. The full list of significant taxa is provided in **Supplementary Table S1**.

### Effect of Maltitol Gum on the Supragingival Plaque Microbiota

**FIGURE 2:**
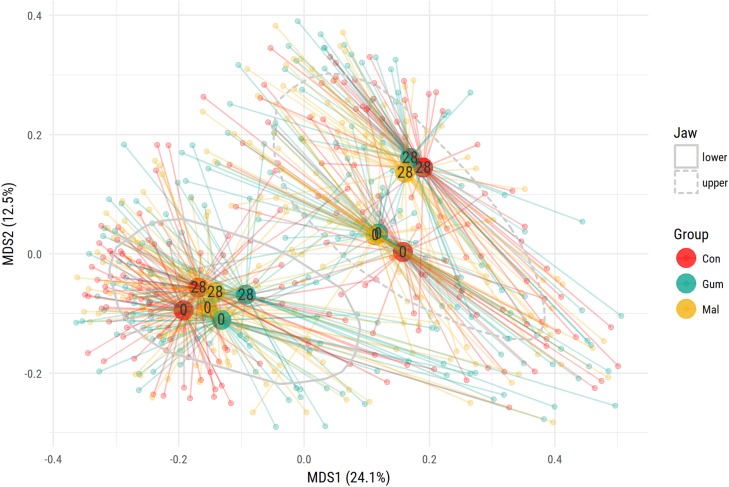
Multidimensional scaling (MDS) ordination plot of supragingival plaque samples. The study groups are indicated by the three colors. For each group, at baseline (0) or at day 28, the centroid value was calculated. The centroid value is shown by the larger dot. Upper buccal plaque microbiota composition is significantly different from the lower lingual microbiota. The gray lines encircle 75% of data points.

To examine the possible effects of the maltitol gum and the gum-base intervention, multidimensional scaling (MDS) was used (**Figure 2**). In both the lower-lingual and upper-buccal plaque samples, a significant (PERMANOVA *P* < 0.05) shift was apparent between the two observation time points, irrespective of the study group. This was most pronounced for the upper buccal plaque microbiota. At the phylum level, the upper buccal microbiota revealed an increase in the abundance of Actinobacteria (19.1–32.4%) and a decrease in Fusobacteria (9.0–2.0%), Bacteroides (14.0–10.3%), and Firmicutes (26.4–24.8%). For the lower-lingual plaque, the phylum-level changes were more modest. The abundance of Actinobacteria increased from an average of 12.0 to 15.8%. We found a decrease in the average abundance of Fusobacteria (28.1–27.3%), Bacteroides (20.6–18.6%), and Firmicutes (18.0–17.8%).

For both the upper-buccal and lower-lingual plaque microbiota, we observed a decrease in abundance of *Haemophilus*, *Leptotrichia*, and *Veillonella*, and an increase in *Aggregatibacter* and *Corynebacterium*.

PERMANOVA analysis showed that there were no significant differences in overall community composition between the three intervention groups at baseline (d0) or at day 28. To identify potential changes in abundance of specific taxa, we used paired nbreg analysis (*P* < 0.05), through which we compared groups, taking into account temporal changes for each individual volunteer.

To examine the effects of gum chewing, we compared the plaque microbiota of the control group with the gum-base group. Effects were found to be weak, suggesting that chewing had a very modest impact on the plaque microbiota. For the lower lingual plaque, the gum base group showed in comparison to the control group significantly higher levels of *Actinomyces* sp., HOT 525, and lower levels of *Prevotella denticola*, HOT 291, *Leptotrichia buccalis*, HOT 563, and *Prevotella* sp., HOT 314. For the upper-buccal plaque, we found lower levels of *Campylobacter concisus*, HOT 575, *Lachnoanaerobaculum umeaense*, HOT 107, and *Eikenella corrodens*, HOT 577 and higher levels of *Dialister invisus*, HOT 118.

**FIGURE 3:**
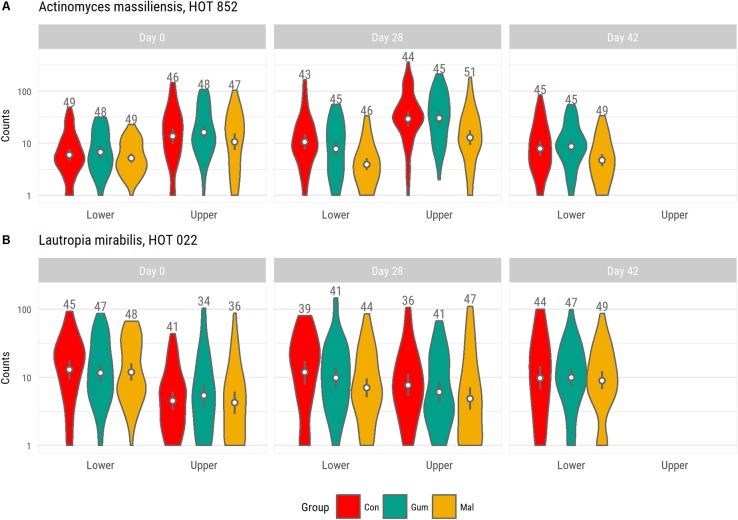
Violin plot shown abundance distribution of *Actinomyces massiliensis* HOT 852 **(A)** and *Lautropia mirabilis HOT* 022 **(B)** for each group. Both species were identified as having significantly lower levels in the maltitol group at day 28 compared to the gum base group. For the upper-buccal plaque microbiota, we also noted lower levels in the maltitol group compared to the gum-base group but these differences were not significant.

To examine the effects of maltitol, we compared the gum-base group microbiota at day 28 with that of the maltitol gum group. For the lower-lingual plaque microbiota, significant (<0.05) differences related to a lower abundance of: *Lautropia mirabilis HOT* 022, *Actinomyces* sp., HOT 170, *Actinomyces massiliensis* HOT 852, and several of *Leptotrichia* species, including *Leptotrichia goodfellowii*, HOT 845, *Leptotrichia shahii* HOT 214, in the samples of the maltitol group compared to the gum base group (**Figure 3**). We found an elevated abundance of *Streptococcus* sp., HOT 056. At day 42, 2 weeks after completion of the gum intervention, levels of *Actinomyces massiliensis*, HOT 852 were still significantly lower than the gum base and the control group.

For the upper-jaw buccal plaque microbiota, we found lower levels of *Actinomyces*_sp., HOT 170, in the maltitol group than in the gum-base group and higher levels of *Streptococcus* sp., HOT 056, *Actinomyces* sp., HOT 180, and *Selenomonas noxia*, HOT 130.

### Maltitol Specifically Inhibits Growth of *Actinomyces* Species *in Vitro*

The results obtained *in vivo*, suggested a possible impact of maltitol on the relative abundance of *Actinomyces* species. We therefore decided to evaluate the possible effect of maltitol on *Actinomyces* growth *in vitro*. For this we performed cultivation assays of *A. naeslundii, A. oris, A. johnsonii, A. massiliensis, A. gerencseriae, A. dentalis*, and *A. israelii* in the presence of 0, 0.25, 0.5, 1, and 2% Maltitol. Growth was assessed qualitatively by daily visual inspections and quantitatively by quantitative PCR at day 7. These studies revealed that growth of *A. massiliensis* and *A. johnsonii* was inhibited in a concentration-dependent manner, showing full inhibition in the presence of 1% maltitol. Growth of *A. israelii* was partially inhibited at maltitol concentrations of 1% and higher. Growth of *A. naeslundii* and *A. gerencseriae* were only slightly impaired at the highest concentration tested. For other species tested, no growth inhibition was observed (**Table 2**).

**Table 2:** *In vitro* growth inhibition of *Actinomyces* species by maltitol.

	*A. naeslundii*	*A. oris*	*A. johnsonii*	*A. massiliensis*	*A. gerencseriae*	*A. dentalis*	*A. israelii*
No maltitol	++	++	++	++	++	++	++
0.25% Maltitol	++	++	++	++	++	++	+
0.5% Maltitol	++	++	++	++	++	++	+
1% Maltitol	++	++	--	--	++	++	+/-
2% Maltitol	+	++	--	--	+	++	+/-


**Actinomyces species were grown anaerobically in SAB medium in the presence of different concentrations of maltitol. After 7 days of incubation, DNA was extracted and the bacterial levels were determined by qPCR. Growth was assessed relative to the control samples to which no maltitol was added: 100% (++), 75% (+), 50% (+/-) 25% (-), 0% (--). While full inhibition was observed for A. johnsonii and A. massiliensis at maltitol concentrations >1%, partial growth inhibition was observed for A. israelii, and limited inhibition for A. naeslundii.**

### Transcriptional Response of *A. massiliensis* Upon Maltitol Exposure

**FIGURE 4:**
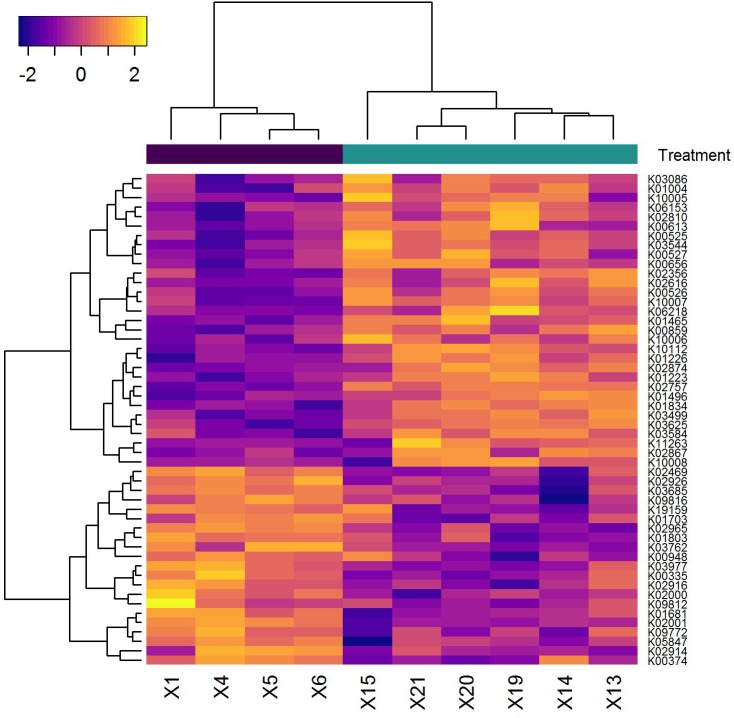
Heatmap of genes (horizontal axis) that showed a significant difference in transcription level in the samples analyzed (vertical axis). The pseudo-coloring of the heatmap is based on the expression levels after Variance Stabilizing Transformation. Samples indicated in black are from the medium control. Those shown in green are the samples following maltitol exposure.

To examine the effects of maltitol at a cellular level, we analyzed the transcriptional response of *A. massiliensis* biofilms after a 60-min exposure to 2% maltitol. Fifty-seven genes were identified with significantly (adjusted *P* < 0.05) lower expression and seventy-two genes with a significantly higher expression level in the maltitol exposed cultures than in the unexposed control (**Figure 4** and **Supplementary Table S2**). We noted a transcriptional differences in a number of genes encoding proteins in metabolism. Among the genes with higher expression, two genes were identified encoding members of the phosphoenolpyruvate-dependent sugar phosphotransferase system (PTS). The first (ORF 1337) annotated as a PTS beta-glucoside transporter subunit IIABC, was adjacent and in the same reading orientation of another upregulated gene encoding an alpha,alpha-phosphotrehalase (ORF 1338). In addition, increased transcription levels were found for a second PTS beta-glucoside transporter subunit IIBCA encoding gene (ORF 1035). Directly upstream of this PTS gene and in the same reading orientation, two significantly upregulated genes were found: the first, ORF 1036, encoding a small transmembrane protein, and the second, ORF 1037, encoding an inorganic phosphate transporter. Lower expression was also found for a number of genes related to metabolic functions. This included a co-localized gene pair encoding a triosephosphate isomerase and a phosphoglycerate kinase, and a gene for an aconitate hydratase, an enzyme that has a central role in the citric acid cycle. Several genes encoding glycine/betaine transporters showed differential expression levels. While we detected an increase in expression of a gene (ORF 1347) encoding a Betaine/Carnitine/Choline Transporter (BCCT) family, we detected lower expression levels of a cluster of four genes (ORF 443–446) encoding a glycine/betaine ABC transporter. Lower expression was also observed for the small conductance mechanosensitive ion channel family MscS encoding gene (ORF 2791). Several genes related to cell wall and attachment functions were altered in expression level. Elevated expression was detected for a gene encoding a Polysaccharide Deacetylases (ORF 1115) and a LytR family transcriptional regulator (ORF 1120), previously implicated in the attachment of anionic polymers to peptidoglycan. Lower expression levels were detected for two adjacent genes, one encoding a collagen-binding surface protein, the other a fimbrial protein (ORF 1144, 1145). Maltitol exposure also led to lower expression levels of a number of ribosomal proteins (ORF 1150–1153), perhaps indicative of lower growth rates.

## Discussion

This study aims to establish the effects of frequent consumption of maltitol-sweetened chewing gum on the supragingival plaque microbiota composition in healthy volunteers. In the study, buccal plaque of the upper-jaw molars was studied independently from plaque collected at the lingual sites of lower jaw molars. While samples from the same individuals were taken at the same time point, the upper-jaw buccal and lower-jaw lingual microbiota displayed a difference. The bacterial levels determined in the lower lingual plaque samples were higher than in the upper buccal samples. Also, the upper buccal and lower lingual plaque microbiota displayed clear compositional differences. While the upper buccal microbiota was dominated by aerotolerant saccharolytic species, the lower lingual plaque was dominated by anaerobic proteolytic species. The anatomical differences between the sites are likely to affect the local physicochemical conditions. Lower jaw lingual surfaces indeed have been demonstrated to have greater accumulations of both hard and soft deposits and more bleeding on probing than other areas of the mouth. The direct vicinity of buccal mucus of the upper buccal plaque samples may promote saccharolytic properties of the plaque biofilm. Likewise, the lower lingual surfaces may be exposed to saliva more directly and for a longer duration, resulting in higher pH values and an excess of nutrients. Sugar measurements following the intake of sugar-containing food products indeed have revealed a non-uniform distribution of sugar in the mouth, with particularly low concentrations at the lingual surfaces of the lower incisors and the buccal surfaces of the upper molars (Macpherson and Dawes, 1994). Clearance was also most rapid from these sites.

Another source for differences in plaque abundance and composition may be due to differences in abrasive forces experienced between these sites. The differences in microbiota composition of supragingival plaque at different anatomical sites has received little attention in microbiota studies, often lacking specific information on the sites selected for sampling or occasionally pooling both lingual and buccal plaque samples (Keijser et al., 2008; Utter et al., 2016; Xiao et al., 2016).

We found a moderate-to-strong correlation in the plaque abundance of the lower lingual plaque samples when comparing samples taken at day 0 with those taken at day 28. This correlation may relate to previously described properties of “heavy” and “light” plaque formers (Simonsson et al., 1987). We did not find such correlation for the upper buccal plaque levels, nor did we observe this when comparing bacterial levels between upper-buccal and lower-lingual plaque. Whether these differences relate to previously reported differences in bacterial aggregation between whole and parotid saliva and between individuals characterized as “light” and “heavy” plaque formers is unclear (Carlén et al., 1996).

During the course of the study, we noted a significant shift in the plaque microbiota, being most pronounced for the upper buccal samples. We noted a significant decline in the bacterial levels in upper buccal samples taken at day 28 as well as a number of microbial changes, including a decline in *Fusobacterium* and *Leptotrichia* species. This suggests the presence of more mature plaque samples at the start of the study compared to those harvested at day 28. This shift was observed irrespective of the group regimen the participants were assigned to. The cause of these differences is not clear. The time of sampling was standardized and performed in the morning by the same examiner. All volunteers were instructed not to brush teeth for 24 h and the duration of plaque accumulation was recorded for each participant in relation to the moment of their clinical visit. It should be noted that all volunteers received a standardized toothbrush and toothpaste upon the start of the study. It cannot be ruled out that the changes in upper buccal plaque abundance and plaque microbiota composition may have been caused by improved dental cleaning efficiency effectuated by the standardized brush and toothpaste. In addition, improved oral hygiene by participants following inclusion in clinical studies has also been described (Hawthorne effect) (McCarney et al., 2007).

By comparing the control group with the gum-base group, we could show that chewing gum five times daily for 28 days had a very minor effect on the composition of the dental plaque microbiota. Statistically significant differences were detected in the abundance of phylotypes between the control group and the gum base group but the differences in abundance levels between the groups were generally small and not always observed in both gum base and maltitol gum groups. In a randomized controlled study with a 3-week duration using a split-mouth model regarding experimental gingivitis, it was shown that in circumstances where regular brushing is performed, no effect of chewing gum was observed on bleeding and plaque scores. In the absence of brushing, chewing xylitol or maltitol gum provided a significant inhibitory effect on gingivitis scores compared to chewing gum base. The difference when compared to the group not using gum was not significant (Keukenmeester et al., 2014).

Maltitol-sweetened gum in the presented study had a very specific effect on a small number of members of the supragingival plaque ecosystem. The strongest effects of maltitol-sweetened gum were observed for *A. masiliensis* and *Lautropia mirabilis* in the lower lingual plaque microbiota. Interestingly, the relative abundance *A. masiliensis* was still lower at day 42, 2 weeks after completion of the intervention. In a recent study, the effects of frequent consumption of maltitol gum during 2 weeks on the interproximal plaque microbiota (dental plaque between teeth) was examined by 454 pyrosequencing (Prosdocimi et al., 2017). The study involved a group of 20 healthy control subjects and 40 subjects with active caries. In line with our results, this study showed that 2 weeks of maltitol gum or gum base had little impact on the microbiota composition as a whole. The study did demonstrate a decline in the relative abundance of a number of *Actinomyces* species. It is important to note that the taxa impacted in this work differed from those identified in our study. It is unclear whether this relates the differences between the niches samples (interproximal plaque or supragingival plaque), or to differences in sequencing platform. Previous studies have revealed dramatic differences in the distribution of that bacteria that differ by as little as a single rRNA nucleotide across habitats of the oral ecosystem. It is unfortunate that in the study of Prosdocimi et al. (2017), interproximal plaque samples of multiple elements from both the upper and lower jaw were pooled, impairing the ability to reveal potential differences between anatomical sites. By *in vitro* cultivation, we could indeed demonstrate that the growth of *A. massiliensis* and *A. johnsonii* is impacted by maltitol. Full growth inhibition was achieved at a concentration of 1%. For the other *Actinomyces* species tested, no or minimal growth inhibition was observed at this level. Transcriptional studies provided insight in the response of *A. massiliensis* biofilms upon exposure to 2% maltitol. While the levels of maltitol applied was prohibitory for growth, the bacterial response was relatively small. First of all, exposure to maltitol effectuated a number of metabolic changes. Increased transcription was found in two genes annotated as PTS beta-glycoside transporters. Lower expression was observed for a number of genes for enzymes that play a central role in carbon metabolism, potentially impacting glycolysis, pentose phosphate pathways, and the citric acid cycle. It is unclear what the possible role of these beta-glucoside PTS glycoside genes is in the uptake of maltitol in *Actinomyces*, and the possible involvement of the alpha,alpha-phosphotrehalase located adjacent to one of the identified PTS genes and of which the expression was elevated as well. The co expression of the transporters and hydrolases may allow for hydrolysis of maltitol upon uptake yielding (phospho-)glucose and sorbitol. This resembles systems identified in other bacterial species, also showing overlapping functions between trehalose uptake and hydrolysis with maltitol (Thompson et al., 2001; Andersen et al., 2012; Ampomah et al., 2013). While glucose would be readily available for metabolism, fermentation of sorbitol in *A. viscosus* and *A. naeslundii* would involve a sorbitol-specific NAD(P)-dependent alcohol dehydrogenase, converting the released sorbitol into fructose (Kalfas et al., 1994; Takahashi et al., 1994). It is noteworthy that while the gene is present on the genome of a number of *Actinomyces* species, translated BLAST analysis suggested that the gene is absent in *A. massiliensis* (data not shown). Future studies are required to establish possible metabolic differences in maltitol and sorbitol fermentation between *Actinomyces* species.

We also noted significant changes in the expression of genes of which the products are involved in osmotic regulation. We found lower expression levels of a gene cluster encoding a glycine betaine ABC transporter and a gene for the small conductance mechanosensitive ion channel (MscS). This coincided with higher expression levels of a BCCT family betaine/carnitine transporter. The transport of organic solutes such as glycine betaine is central in bacterial osmoregulation. We also detected increased expression of a gene encoding a polysaccharide deacetylase, which in Bacillus species has been shown to play a role in osmotic stability and cell-shape maintenance (Arnaouteli et al., 2015). The changed expression levels of osmoregulation-associated proteins may reflect adaptations to changing osmotic conditions. At the same time, the transcriptional changes observed also imply a shift from an ATP-driven process for solute import to one driven by a transmembrane gradient and may perhaps also reflect cellular adaptation to ATP depletion.

A third process that emerged from analysis of the transcriptional response following maltitol exposure relates to lower expression levels of surface associated aggregation proteins such as fimbriae. As *A. massiliensis* growth appears to be impaired in the presence of maltitol, changes in cellular adhesion may perhaps relate to a shift from biofilms toward planktonic growth. While the transcriptional response upon maltitol exposure may provide indications toward the molecular impact of the polyol on metabolism and osmotic regulation of *Actinomyces* biofilms, further studies are required to establish these.

The clinical significance of these findings is unclear. Little is known of the role of *Lautropia mirabilis* in the supragingival biofilm or its relation with disease. Structural studies have suggested that *Lautropia mirabilis* forms specific cauliflower-like structures within the multispecies biofilm but its role in plaque biofilm formation is not well understood. *Actinomyces* species have been studied extensively (Kononen and Wade, 2015). Various *Actinomyces* species, have been implicated in (root) caries (Brailsford et al., 1999; Tanner et al., 2011; Kononen and Wade, 2015). *Actinomyces* species are primary colonizers and have a prominent role in the supragingival biofilm structure (Mark Welch et al., 2016). Although *A. massiliensis* is a dominant *Actinomyces* species within the lower-lingual plaque microbiota, we only detected limited impact on the lower lingual plaque microbiota composition in volunteers after 24 h of plaque accumulation. Given the impact of maltitol on prominent members of species that act as primary colonizers of supragingival dental surfaces, it seems worthwhile to investigate the possible effects on the early dental plaque microbiota development.

## Conclusion

This study has shown that daily use of chewing gum in healthy individuals has little impact on the supragingival plaque microbiota composition. Frequent use of maltitol gum resulted in selective inhibition of a number of bacterial members of the supragingival plaque microbiota, some of which are recognized as early colonizers of dental surfaces. To what extent this influences the dynamics of dental plaque accumulation, the functional properties of the plaque biofilm or the impact this has on clinical parameters of oral health remains to be established.

## Availability of Data and Materials

The 16S ribosomal sequence and RNA-seq datasets generated and analyzed during the current study will become publicly available in the European Nucleotide Archive (ENA) upon acceptation of the paper. http://www.ebi.ac.uk/ena.

## Author Contributions

BK, DS, FvdW, and CT designed the experiments and wrote the study protocols. DS and FvdW were responsible for patient recruitment and clinical data collection. RM, BK, JK, and MO were responsible for sample preparation and 16S-rRNA sequencing and transcription studies. BK, LvT, and TvdB were responsible for bioinformatics processing and statistical analyses. BK wrote the paper. All authors significantly contributed to interpreting the results, critically revising the manuscript for important intellectual content, and approving the final manuscript.

## Conflict of Interest Statement

BK and JK have previously received research grants from Wrigley and Cargill. CT is employed by Roquette. FvdW, DS and their research team at ACTA have previously received research grants from Wrigley, Roquette, Procter & Gamble, Sara Lee, Sunstar, and Unilever. The other authors declare that the research was conducted in the absence of any commercial or financial relationships that could be construed as a potential conflict of interest.

## Footnotes

1 www.random.org

2 www.homd.org

## Supplementary Material

**FIGURE S1 |** Study flow diagram.

**TABLE S1 |** Table provides a list of significant MED nodes that were found to be differentially (*P* < 0.05) abundant between upper-jaw buccal and lower-jaw lingual supragingival plaque, and differentially (*P* < 0.05) abundant between study groups for the upper jaw buccal and lower jaw lingual plaque microbiota.

**TABLE S2 |***Actinomyces massiliensis* genes identified with significant differences in expression in biofilms after between the medium control and exposure to medium supplemented with 2% of maltitol. The column values represent the ORF number, as found in the contig files of *Actinomyces massiliensis* F0489 (ACCESSION NZ_AKFT00000000), the description found as in the GenBank files, the average of the normalized counts taken over all samples (baseMean), log2 fold change between the groups (log2FoldChange), the standard error of the log2FoldChange estimate (lfcSE), Wald statistic (stat), Wald test *p*-value (pvalue), the Benjamini-Hochberg adjusted *p*-value (padj).
