# Supplementary figures and images for: The Impact of Maltitol-Sweetened Chewing Gum on the Dental Plaque Biofilm Microbiota Composition
Source: Front Microbiol. 2018 Mar 6;9:381. doi: 10.3389/fmicb.2018.00381 (PMC5845675; doi:10.3389/fmicb.2018.00381)

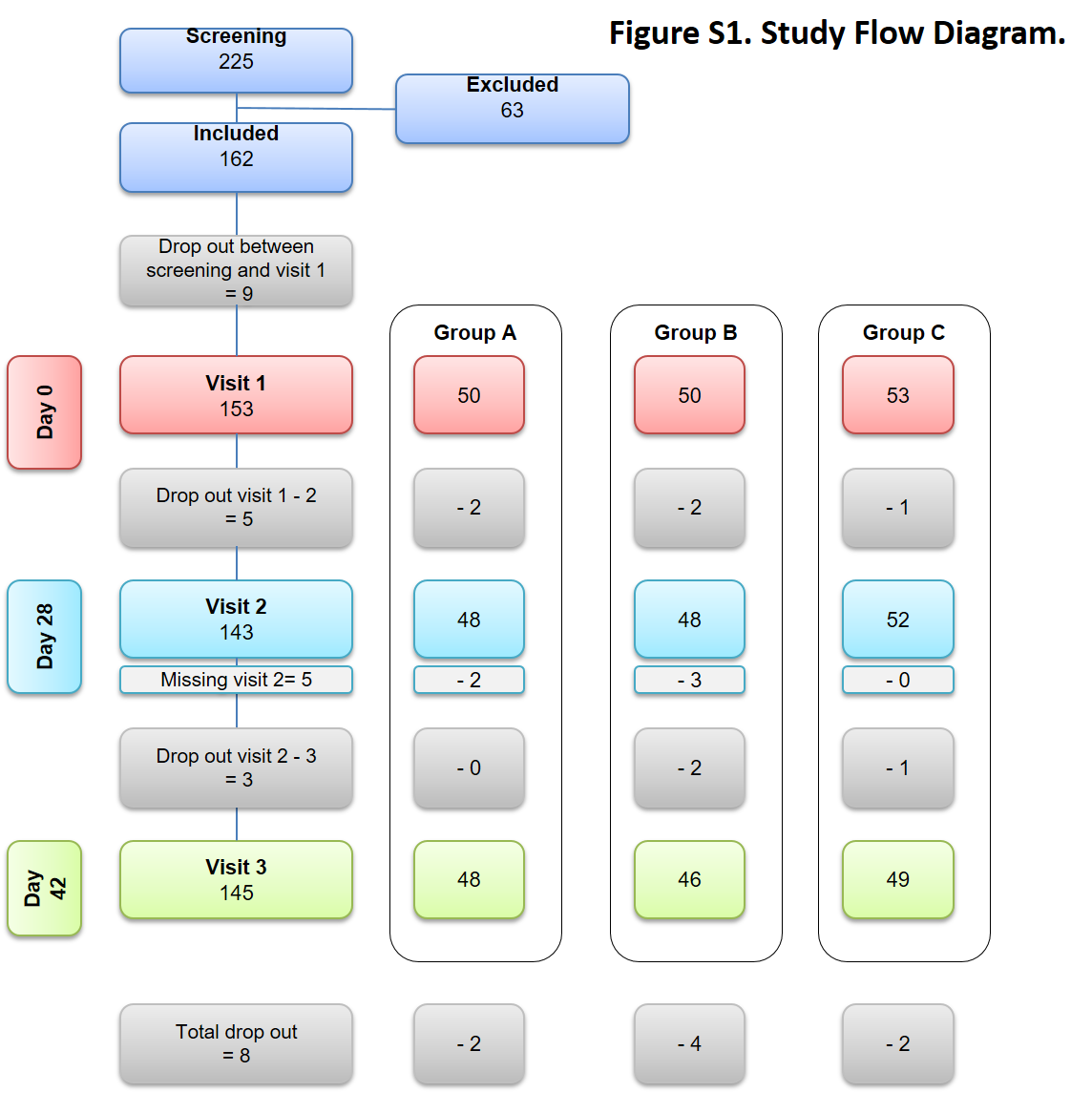

Supplement: Supplementary file 1 [file Image_1.PNG]
